# Genome‐wide meta‐analysis and functional validation identified MS4A6A/Ms4a6d critical role in Alzheimer's disease

**DOI:** 10.1002/alz70855_096874

**Published:** 2025-12-23

**Authors:** Haishan Jiao

**Affiliations:** ^1^ Huashan Hospital, Fudan University, Shanghai, Shanghai, China

## Abstract

**Background:**

Genetic variations in the MS4A6A gene have been implicated in modifying the risk of Alzheimer's disease (AD). Earlier research suggested a potential disease‐promoting role of MS4A6A in AD, as its expression levels were found to correlate with the severity of clinical neuropathology. Despite these observations, the underlying mechanisms connecting MS4A6A to AD pathogenesis remain unexplored experimentally.

**Method:**

We performed a meta genome‐wide association analysis with 734,121 subjects to examine the associations between polymorphisms of *MS4A6A* with AD risks. In addition, we analyzed the correlation between MS4A6A and AD‐related cerebrospinal fluid biomarkers from our own cohort. Furthermore, we for the first time generated a Ms4a6d deficient APP/PS1 model, and systematically examined pathological changes using high‐resolution microscopy, biochemistry, and behavioral analysis.

**Result:**

We identified several novel MS4A6A mutations associated with variations in Alzheimer's disease (AD) risk, some of which were linked to altered β‐amyloid levels in cerebrospinal fluid. In APP/PS1 mice deficient in Ms4a6d, microglial function was significantly compromised, leading to impaired amyloid clearance, increased plaque accumulation, less compact plaque structures, and exacerbated synaptic damage. Additionally, Ms4a6d deficiency intensified inflammatory responses in microglia and astrocytes through dysregulation of the NF‐κB signaling pathway.

**Conclusion:**

Our findings reveal that Ms4a6d deficiency suppresses neuroprotection and worsens neuroinflammation. Thus, the elevated MS4A6A levels in AD are likely compensatory and boosting MS4A6A could be an effective treatment.

